# Simple and Efficient Methods for Enrichment and Isolation of Endonuclease Modified Cells

**DOI:** 10.1371/journal.pone.0096114

**Published:** 2014-05-05

**Authors:** Branden S. Moriarity, Eric P. Rahrmann, Dominic A. Beckmann, Caitlin B. Conboy, Adrienne L. Watson, Daniel F. Carlson, Erik R. Olson, Kendra A. Hyland, Scott C. Fahrenkrug, R. Scott McIvor, David A. Largaespada

**Affiliations:** 1 Department of Genetics, Cell Biology and Development, University of Minnesota, Minneapolis, Minnesota, United States of America; 2 Center for Genome Engineering and Institute of Human Genetics, University of Minnesota, Minneapolis, Minnesota, United States of America; 3 Masonic Cancer Center, University of Minnesota, Minneapolis, Minnesota, United States of America; 4 Discovery Genomics, Inc, Minneapolis, Minnesota, United States of America; 5 Department of Pediatrics, University of Minnesota, Minneapolis, Minnesota, United States of America; 6 Department of Animal Science, University of Minnesota, Minneapolis, Minnesota, United States of America; 7 Recombinetics, Inc., Saint Paul, Minnesota, United States of America; Southern Illinois University School of Medicine, United States of America

## Abstract

The advent of Transcription Activator-Like Effector Nucleases (TALENs), and similar technologies such as CRISPR, provide a straightforward and cost effective option for targeted gene knockout (KO). Yet, there is still a need for methods that allow for enrichment and isolation of modified cells for genetic studies and therapeutics based on gene modified human cells. We have developed and validated two methods for simple enrichment and isolation of single or multiplex gene KO's in transformed, immortalized, and human progenitor cells. These methods rely on selection of a phenotypic change such as resistance to a particular drug or ability to grow in a selective environment. The first method, termed co-transposition, utilizes integration of a *piggyBac* transposon vector encoding a drug resistance gene. The second method, termed co-targeting, utilizes TALENs to KO any gene that when lost induces a selectable phenotype. Using these methods we also show removal of entire genes and demonstrate that TALENs function in human CD34^+^ progenitor cells. Further, co-transposition can be used to generate conditional KO cell lines utilizing an inducible cDNA rescue transposon vector. These methods allow for robust enrichment and isolation of KO cells in a rapid and efficient manner.

## Introduction

Reverse genetic approaches in human cells have proven fruitful for understanding conditions such as cancer and neurodegenerative diseases. However, even with the multiple forms of mRNA knock down (KD) available, such as small hairpin RNA (shRNA), small interfering RNA (siRNA), and microRNAs (miRNA) there are still not simple and reliable methods to completely knockout (KO) gene function to eliminate all protein expression, as is observed in many human cancers. Moreover, shRNA technologies vary in efficacy among cell lines, can be silenced by the host cell, and need to be maintained under drug selection to ensure continued target knockdown, a drawback that critically impairs *in vivo* xenograph studies. Thus, it may be necessary to mutate and inactivate, or completely remove, an endogenous loci to ablate protein levels to model diseases where complete loss of gene function is observed. Moreover, as new candidate cancer genes are being rapidly identified by whole genome sequencing efforts and forward genetic screens it is important that robust methods to completely KO gene function become more accessible and efficient to study these genes functionally [1]–[5]. This is also true of gene therapy studies to model or treat genetic diseases, where eliminating endogenous gene expression is critical, such as targeting *CCR5* in T-cell progenitors for HIV treatment [6].

The recent advent of TALENs, and similar targeted nucleases such as the CRISPR system, offer a reliable and cost effective avenue for targeted gene KO for genetic studies and therapies conceivably obtainable for any lab[7]–[9]. Though many labs may not have the expertise in nuclease design or implementation to consistently achieve high rates of modification for their gene of interest (GOI). This combined with the fact that numerous clones must be isolated and analyzed to identify KO clones demonstrates that simple enrichment and isolation methods are needed in order to expand the use of designer nucleases to generate KO cell lines for research. Moreover, isolation of nuclease modified cells intended for therapeutic applications could also be improved by the use of enrichment methods. However, nearly 4 years after the advent of TALENs there is still a lack of simple and efficient methods for isolating KO cell lines generated by targeted nucleases[10].

There have been a limited number of articles demonstrating enrichment of nuclease modified cells, these methods typically rely on fluorescence activated cell sorting (FACS) using a surrogate nuclease reporter plasmid or having the nucleases linked physically or transcriptionally to a fluorescent protein in some manner [11],[12]. Unfortunately, cells that have undergone FACS are exposed to intense lasers and high hydrostatic pressure, reducing their viability, in addition to the need for a FACS machine[13]. Further, the use of a surrogate nuclease reporter plasmid requires the construction of a new reporter vector for every intended nuclease target site. Importantly, these methods do not allow for selection of enriched cells to generate individual clones for analysis. This is a large impediment for functional studies of gene loss in cancer studies using transformed cell lines.

An ideal method for enrichment and isolation of nuclease modified cells would be one that functions in nearly all cell types, uses a universal construct, relies on a simple and effective phenotypic selection to easily generate clones, and consistently increase the frequency of generating nuclease modified clones to expedite identification of KO clones. To this end, we developed and validated simple and efficient, single step methods for enrichment and isolation of KO mammalian cells. These methods rely on selection of a phenotypic change such as resistance to a particular drug or ability to grow in a selective environment, such as soft agar.

The first method, termed co-transposition, uses co-transfection of TALENs with a *Piggybac* (*PB*) transposon carrying a drug selectable marker to allow for enrichment and isolation of TALEN modified cells for genetic studies[14]. We validated this method using 12 different TALEN pairs targeting tumor suppressor genes, proto-oncogenes, and seemingly inert genes with increased gene modification compared to unselected TALEN treated cells observed in nearly all cases. The second method, termed co-targeting, uses TALENs to target a gene that allows for phenotypic selection of modified cells. We demonstrate two unique selectable phenotypes. Targeting the tumor suppressor *PTEN* in immortalized human Schwann cells induces anchorage independent growth in soft agar, which allows for simple isolation of cells successfully targeted for KO of *PTEN* and other GOI co-targeted with TALENs. Lastly, in an effort to increase gene modification for cells potentially intended for therapeutic purposes, we successfully implement co-targeting of *HPRT* using CD34^+^ human cord blood progenitor cells to enrich for modification of therapeutically relevant genes. Our results demonstrate that the use of co-transposition and co-targeting allows for rapid generation of TALEN modified cells for research and therapeutic applications.

## Materials and Methods

### Vector design and assembly

Candidate TALENs were designed using TALE-NT (https://boglab.plp.iastate.edu/node/add/talen) or using previously described and validated target sites (Table S1)[15]. From the list of candidate TALENs generated using TALE-NT, optimal TALENs were chosen and constructed based on RVD content, spacer length, and binding site length based on previous TALEN publications and our own experiences with TALENs[8],[9],[16],[17]. Briefly, stretches of NG/NI RVDs were avoided, high HD content, spacer and binding site length ranging from 15–18; though we have developed functional TALENs that go against these general parameters. TALENs were assembled using Golden Gate cloning as previously described[18]. The truncated Δ152+63 TALEN backbone and ‘cold'shock’ method used have been previously described[8],[19],[20]. The *piggyBac* transposon PB-CAGG-Luciferase-IRES-GFP-PGK-Puro was generated by LR clonase reaction (Invitrogen) of pENTR221-Luciferase with PB-CAG-DEST1-IRES-GFP-PGK-Puro as previously described[21]. The conditional rescue *PB* vector was generated by Cre recombinase retrofitting of R26 (-) TRE-DEST1 with PB-EF1A-rtTA-IRES-Puro as previously described[22]. TALEN resistant cDNAs (TR-cDNAs) were generated by performing inverse PCR of pENTR221-cDNA vectors engineering silent nucleotide changes in both forward and reverse PCR primers. TR-cDNAs were then transferred to PB-TRE-DEST1-EF1A-rtTA-IRES-Puro by standard LR Clonase reaction (Invitrogen). The PB-mCAG-DHFR:EGFP transposon plasmid was constructed from the previously described DL2G plasmid[23]. The entire transposon cassette, including mCAGs, DHFR(tyr22)-eGFP, and bovine growth hormone polyA was removed using *XhoI* to *NheI* sites and inserted into analogous sites of PB-MCS (LR5). The CRISPR system was obtained from Addgene[7]. The hCas9 cDNA was PCR amplified and cloned into pENTR1 Gateway vector using SnaBI and XbaI engineered into the PCR primers. An N-terminal Flag tag sequence was also included in the forward primer. The Flag-hCas9 cDNA was then moved to PT3.5-CAGG-DEST using the LR Clonase reaction (Invitrogen). U6-gRNA vectors were produced using inverse PCR of TOPO4-U6-gRNA vector with unique 19 bp target sequences engineered into the forward primer and a common reverse primer, followed by T4 polynucleotide kinase treatment (New England Biolabs) and self-ligation with T4 Ligase (New England Biolabs). Primer sequences can be found in Table S2.

### Cell culture, drug selection, and electroporations

All cells were maintained in DMEM medium supplemented with 10% FBS and 1% Penicillin/Streptomycin. HCT116 cells were purchased from ATCC. 2462-TY and immortalized human Schwann cells have been previously described[21]. All TALEN transfected cells, unless noted otherwise, underwent ‘cold shock’ for 2 days at 30°C after 1 day at 37°C to increase the frequency of gene modification. Electroporations were performed using the NEON electroporation system (Invitrogen) using 100 µL electroporation tips, following manufacturers instructions. One million cells were electroporated with 2 µg of each left and right TALEN vector or 2 µg Flag-hCas9 and U6-gRNA vector with 100 ng of pmaxGFP plasmid (Amaxa) to assess transfection efficiency. Co-transposition was performed using an additional 500 ng of *PB* transposon and 500 ng of CMV-*PB7* transposase.

### Cel-I assay and analysis

CEL-I assays were performed as previously descried[24]. Briefly, after electroporation of TALEN encoding plasmids and incubation for 3 days genomic DNA was extracted using DNeasy Blood and Tissue Kit (Qiagen), following manufacturers instructions. PCR amplicons were generated spanning the TALEN binding site using Accuprime Taq HF (Invitrogen) using the following PCR cycle: initial denaturation at 95°C for 5 min; 40× (95°C for 30 sec, 55°C or 60°C for 30 sec, 68°C for 40 sec); final extension at 68°C for 2 min. PCR amplicons were denatures and annealed as follows: 95°C for 5 minutes, 95–85°C at −2°C/s, 85–25°C at −0.1°C/s, 4°C hold. Primer sequences can be found in Table S2. Three microliters of the annealed amplicon was then diluted with 6 µL of 1× Accuprime PCR buffer and treated with 1 µL of Surveyor nuclease with 1 µL of enhancer (Transgenomics) at 42°C for 20 min. The reaction was then stopped by the addition of 3 µL of 15% Ficol-400 and 0.05% Orange G solution containing 1 mM EDTA and subsequently run on a standard 10% TBE gel. Percent gene modification was calculated using Image J software as described[24].

### Western blot analysis

Cells were harvested and lysed with modified RIPA buffer (0.5% (vol/vol) NP-40, 50 mM Tris-HCl pH 7.4, 150 mM NaCl, 1 mM EDTA) containing phosphatase inhibitors (Sigma) and a complete mini protease inhibitor pellet (Roche). Cells were incubated on ice for 10 min followed by sonication with 10 pulses at 30% power. *TP53* (2527), *NF2* (6995), *PTEN* (9188), *CCND1* (2978) antibodies were purchased from Cell Signaling Technology. AcV5 (A2980) antibody was purchased from Sigma.

### Knockout sequence analysis

TALEN target site amplicons were amplified as in the CEL-I protocol described above. Purified PCR products were directly sequenced by single pass Sanger sequencing (ACGT, Inc and the University of Minnesota Biomedical Genomics Center).

### Proliferation and soft agar growth assays

Proliferation assays were set up in a 96-well format with 100 cells plated per well. Proliferation was assessed every 24 hours over 6 days by the MTS assay, following manufactures instructions (Promega). Absorbance was read at 490 nm to determine proliferation and 650 nm to account for cellular debris on a BioTek Synergy Mx automated plate reader. Soft agar assays were performed as follows, 6-well plates were prepared with bottom agar composed of 3.2% SeaPlaque Agar (Lonza) in DMEM full media and allowed to solidify before 10,000 cells in top agar (0.8% SeaPlaque Agar in DMEM full media) were plated and allowed to solidify. DMEM full media with 2.5 µg/mL doxycycline was plated over the cells and cells were incubated under standard conditions (5% CO_2_,37°C) for 2 weeks. Top media was removed and cells were fixed in 10% formalin (Fisher Scientific) containing 0.005% crystal violet (Sigma) for 1 hour at room temperature. Formalin was removed and colonies were imaged on a Leica S8 AP0 microscope. 12 images per cell line were taken and automated colony counts were done using ImageJ software. Results shown are a representative example of at least 3 independent experiments.

### Nucelofection of Cd34^+^ cells

Umbilical cord blood (UCB) was purchased from the National Disease Research Interchange. After Ficoll-Paque PLUS (1.077 density, GE Healthcare) gradient separation, UCB mononuclear cells were collected and enriched for CD34^+^ cells using Stem Cell Technologies positive magnetic selection. CD34^+^ cells were cultured overnight with Xvivo10 serum free media with gentamicin (Lonza), containing SCF, TPO, IL-3 and Flt3L (30 ng/ml). Subsequently, CD34^+^ cells from each cord were mixed with autologous CD34^−^, similarly cultured overnight (10–20% CD34^+^ cells) at a concentration of 1×10^6^ cells/0.1 mL of human CD34 cells and nucleofected (Amaxa) with a total of 10 µg TALEN encoding or *piggyBac* transposon/transposase plasmids using program U-08. Transfected cells were immediately transferred to 12-well plates containing 37°C pre-warmed Xvivo10 with human cytokines.

### *In Vitro* culture of Cd34^+^ cells

After five days in liquid culture, with fresh media added on day 2–3, ≥85% of cells expressed CD34, as measured by flow cytometry after staining with anti-human CD34 (clone 4H11, eBioscience). CD34^+^ cells were plated in methylcellulose medium containing human cytokines (HSC005, R&D Systems) in the presence and absence of 5 µg/ml 6-thioguanine (Sigma) or 100 nM methotrexate (Bedford Laboratories) and 5 µM dipyridamole (Sigma). After 12 to 16 days at 37°C, 5% CO_2_, the progenitor colonies containing >50 cells were counted by microscopy and categorized by morphology as either erythroid (BFU-E or CFU-E) or granulocyte/monocytic morphology (CFU-GM) colonies.

## Results

### Co-Transposition using *Piggybac* allows for enrichment and Isolation of talen modified clones

**Figure 1:**
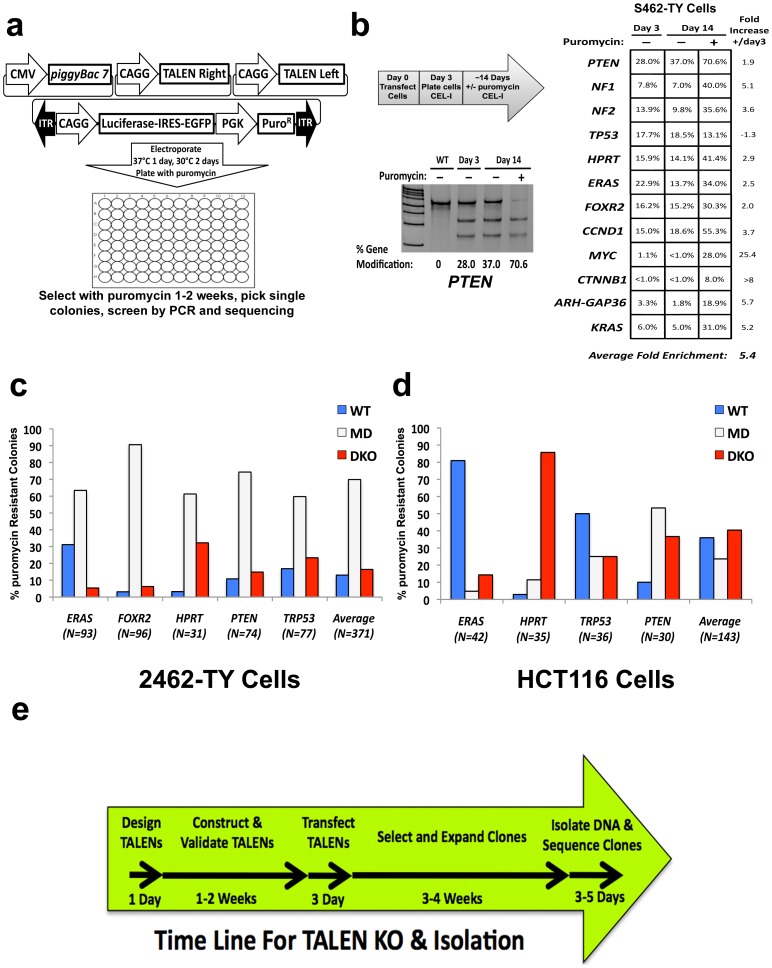
Co-transposition allows for robust enrichment and isolation of TALEN modified cells. (a) Diagram of co-transposition method. Cells were transfected with TALEN plasmids in addition to CMV-PB7 and PB-CAGG-Luciferase-IRES-EGP-PGK-Puro transposon. (b) Co-transposition increases TALEN mediated genome modification in S462-TY cells. Outline of experimental timeline to test co-transposition method for enrichment of modified cells (top left). Example of CEL-I assay results with co-transposition using *PTEN* TALENs (bottom left). Table of co-transposition results using 12 independent TALEN pairs (right). (c) Analysis of number of wild type (WT), mutation detected (MD), and double knockout (DKO) clones isolated using co-transposition in S462-TY and HCT116 cells by direct sequencing. Example chromatograms for these mutation classes can be found in Figure S2. (d) Time line for generating TALEN KO clones using co-transposition beginning with TALEN design and ending with validating isolated clones via sequencing.

As it has been previously reported that cells expressing high levels of nuclease protein produce more DSBs, we hypothesized that a method that enriches for cells transfected with high levels of TALEN encoding plasmid could enrich for TALEN modified cells[12],[20]. Additionally, it would be ideal to select for these cells using a simple drug selection strategy. As *piggyBac* (*PB*) mediated transposon transposition is a relatively inefficient process when transposase expression is limited, i.e. low levels of transposase encoding plasmid, we hypothesized that co-transfection of TALENs with limited *PB* transposase and *PB* transposon encoding a drug selectable marker could allow for both enrichment and isolation of TALEN modified cells (Figure 1a)[25]. We have termed this method co-transposition as TALENs are co-transfected with the *PB* system that undergoes transposition.

To test this hypothesis, we performed co-transposition using 12 independent TALEN pairs with a puromycin encoding *PB* transposon into S462-TY malignant peripheral nerve sheath tumor (MPNST) cells and allowed the cells to grow for 14 days with or without drug selection (Figure 1b). Using the CEL-I assay as a readout, all but one TALEN pair (*TP53*) demonstrated increased gene modification with drug selection; with a fold increase from day 3 to 14 ranging from 1.9–25.4 fold (∼5 fold on average) (Figure 1b). These experiments were performed using a ‘cold shock’ treatment at 30°C as we, and others, have found this increases TALEN activity and subsequent gene modification (Figure S1)[19]. Interestingly, using TALENs to known oncogenes of WNT/Beta-catenin signaling (*MYC* and *CTNNB1*), recently reported to be critical for MPNST cell maintenance, demonstrated near undetectable levels of gene modification in MPNST cells without co-transposition; though these TALENs were previously validated in U2OS osteosarcoma cells and produced robust gene modification by transient transfection (13% and 26% for *MYC* and *CTNNB1*, respectively)[26],[27]. However, when *MYC* and *CTNNB1* were targeted with co-transposition gene modification rates were much higher, demonstrating the power of selection of this system even in a situation where a cell population relies on expression of the target genes.

Next, we implemented co-transposition for cancer gene KO to assess the ability of co-transposition to generate viable KO clones and also determine a rate of TALEN modified clones. Puromycin resistant clones were analyzed by direct sequencing of PCR products of TALEN target sites, which were then classified as wild type (WT), mutation detected (MD), or double knockout (DKO) clones (Figure S2). DKO clones contain identical or near identical bi-allelic insertion or deletion (indels) mutations, where as MD clones are mutated at one or more alleles with different indels. The classification of MD was used in place of cloning and sequencing different alleles as the copy number of target genes in transformed cells is unknown and likely varies dramatically. Moreover, the definitive test of Western blot analysis to determine loss of protein expression should be performed on a panel of isolated MD and DKO clones to identify clones for use in functional studies or biochemical assays, which is substantially cheaper and faster than cloning all alleles of every MD clone identified.

We identified clones of each classification with every TALEN pair tested in both 2462-TY and HCT116 cells with only 14.6% (n = 371) and 39.2% (n = 143) of clones found to be WT, respectively (Figure 1c and d). Though our transfection efficiencies in these immortalized cell lines were consistently over 80%, co-transposition also enriches for nuclease modified cells in poorly transfectable cell lines (data not shown). Moreover, modified cells appropriately expressed EGFP and luciferase, also encoded in the puromycin containing transposon, demonstrating that co-transposition can be used to concurrently engineer modified cells to express multiple heterologous genes (Figure S3). In fact, co-transposition was effective with our recently described RecWay assembled, multigene, transposon vectors containing up to 6 transgenes and ∼30 kb in size (Figure S4)[28]. Co-transposition can also be multiplexed to KO more than one gene at a time and is functional in other transformed and immortalized cell lines (Figure S5). These data demonstrate that co-transposition is a robust method for enrichment and isolation of nuclease modified cells and also allows for concurrent engineering of targeted cells to express numerous heterologous genes, which can be easily performed in less than 2 months (Figure 1e). Moreover, we found that this method is not limited to TALENs but is also highly efficient with the recently described CRISPR system, increasing gene modification ∼5 fold on average with rates ranging from 41.5–55.6% (Figure S6)[7]

### Conditional rescue Co-Transposition for inducible Ko cell lines

**Figure 2:**
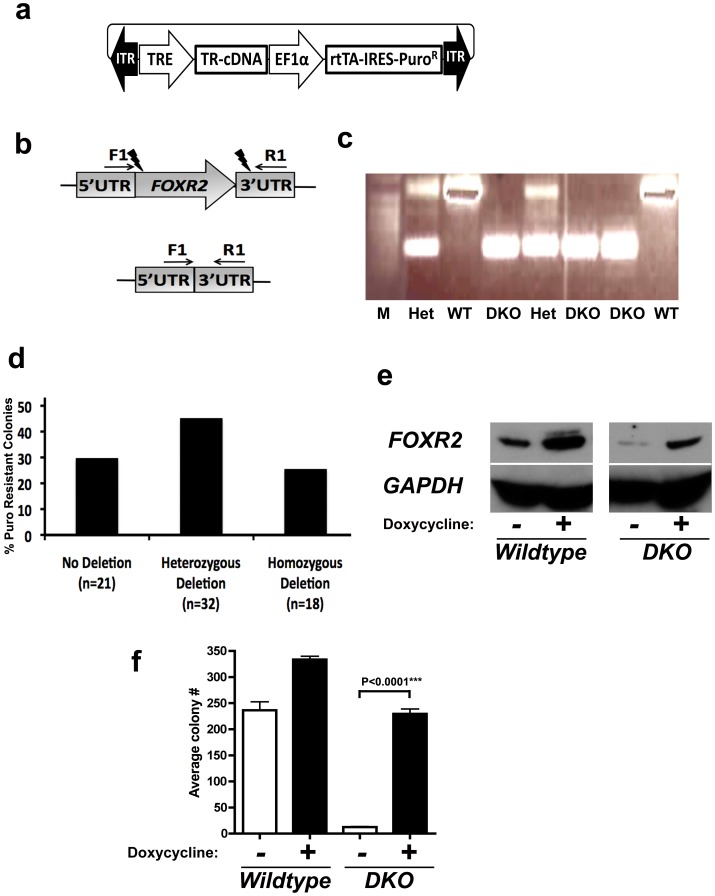
Conditional rescue co-transposition allows for functional inducible knockout cell lines. (a) Diagram of all-in-one doxycycline inducible conditional rescue vector. TALEN resistant cDNA (TR-cDNA) are activated via the ‘dox-on’ rtTA transactivator in the presence of doxycycline. (b) Diagram of proto-oncogene *FOXR2* locus demonstrating the TALEN target sites (indicated by lightning symbols) and primers used to analyze clones for deletion of *FOXR2* locus (indicated by arrows). (c) Representative PCR results from analysis of clones generated using conditional rescue co-transposition targeting deletion of the *FOXR2* locus in S462-TY cells. Molecular weight ladder (M) is also shown. (d) Results of PCR and direct sequencing analysis of 71 clones for whole deletion of one or both *FOXR2* alleles. (e) Example of a functional conditional rescue *FOXR2* wild type and DKO clone via Western blot analysis with and without addition of doxycycline. (f) Functional validation of conditional rescue clones via soft agar colony formation assay of clones shown in (e) in the presence or absents of doxycycline demonstrating induction of colony formation in *FOXR2* DKO clone with addition of doxycycline. Wild type clones underwent co-transposition with both *FOXR2* TALEN pairs but remained unmodified at the *FOXR2* locus. Statistical analyses were performed using two tailed t-test.

When targeting oncogenes using co-transposition it was noted that the number of DKO clones generated was consistently lower compared to experiments where tumor suppressors genes or seemingly inert genes, such as *HPRT*, were targeted (Figure 1c). It is possible that cells where oncogenes were inactivated by TALEN induced mutations were less viable and therefore rarely isolated. Thus, we developed a conditional rescue system to generate inducible KO cell lines of target genes that are addictive oncogenes or essential genes. We hypothesized that supplementing cells continually with target gene expression via cDNA expression could allow for the isolation of viable KO clones. This approach uses an all-in-one doxycycline inducible transposon to express a TALEN resistant cDNA (TR-cDNA) of the target gene in addition to the puromycin resistance gene (Figure 2a). We found that cDNAs can be made TR by introduction of silent mutations at the TALEN target site in the cDNA. We further flanked the TRE-cDNA portion of the vector with LoxP sites to allow for complete removal of the TR-cDNA in addition to being doxycycline regulatable. In an effort to test the conditional rescue system we targeted the recently described proto-oncogene *FOXR2* by removal of the entire gene[21].

To this end, we generated TALENs flanking the entire open reading frame (ORF) of this single exon gene; one targeting just after the ATG start codon and the other just after the stop codon (Figure 2b). We identified numerous clones with heterozygous and homozygous deletion of *FOXR2* by PCR and direct sequencing using 2462-TY cells, all of which demonstrated fusion of the 5′ and 3′ UTRs (Figure 2c,d). We were able to identify conditional rescue DKO clones that dependably induced TR-*FOXR2* cDNA expression upon treatment with doxycycline by Western blot analysis (Figure 2e). It is also known that loss of *FOXR2* in MPNST cells substantially reduces their ability to form colonies in soft agar[21]. Importantly, this functional read out was significantly induced upon treatment with doxycycline and nearly undetectable in the absence of TR-*FOXR2* induction (P<0.0001***, Two-tailed t-test) (Figure 2f).

Co-transposition conditional rescue is not limited to deletion of an entire gene or *FOXR2* as we were able to generate DKO cell lines carrying the corresponding inducible TR-cDNA transposon for *CCND1*, targeting just after the ATG start codon, and demonstrate functional read-outs of significantly enhanced proliferation and growth in soft agar upon activation of TR-*CCND1* with doxycycline (P = 0.0211* and P = 0.0017**, respectively, t-test) (Figure S7). Taken together, these data demonstrate that co-transposition using a conditional rescue transposon vector is a viable option for making conditional KO cell lines.

### Anchorage independent growth induction using Co-Targeting enriches for talen modified cells

**Figure 3:**
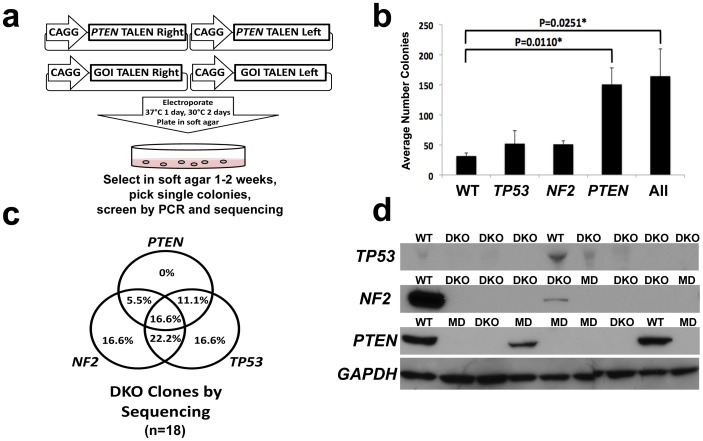
Co-targeting ***PTEN*** allows for robust enrichment and isolation of immortalized human Schwann cells. (a) Diagram of co-targeting *PTEN* method. (b) Number of colonies formed in soft agar following targeting with *PTEN*, *TP53*, or *NF2* TALENs alone or in combination compared to untransfected immortalized human Schwann cells (student t-test). (c) Percent DKO clones based on sequence analysis of soft agar selected clones transfected with all three TALEN pairs (n = 18). 11.4% of these clones were either classified as WT or MD at all three target genes. (d) Western blot analysis of a subset of clones analyzed by sequencing.

Next, we wanted to develop a co-targeting method for enrichment and isolation of TALEN modified clones that would rely on a selectable phenotype. To this end, we utilized co-targeting of genes using TALENs to induce anchorage independent growth (Figure 3a). Using an immortalized human Schwann cell line we implemented TALENs targeting *PTEN*, *TP53*, and *NF2* individually and in combination[21]. Interestingly, when using individual TALENs only *PTEN* targeting significantly induced colony formation compared to untargeted controls, indicating that *PTEN* loss is a strong driver of anchorage independent growth in this cell type (Figure 3b) (P = 0.0110*, t-Test).

When all three TALEN pairs were multiplexed, 16.6% of analyzed clones were DKO at all three targets by direct sequencing (Figure 3c). Moreover, upon Western blot analysis an additional 20% were DKO for all targets at the protein level as many MD clones were presumably KOs with different indels (Figure 3d). DKO clones containing residual protein could be from small in-frame indels that may or may not disable protein function. Importantly, in this single experiment we generated every combination of gene mutation, i.e. heterozygous and homozygous mutations, from analyzing a small number of clones (n = 18). These data demonstrate that co-targeting is a powerful method for modeling *de novo* transformation and studying combinations of gene mutations in immortalized human cells.

### Induction of 6-Thioguanine resistance enriches for talen modified Cd34^+^ progenitor cells

**Figure 4:**
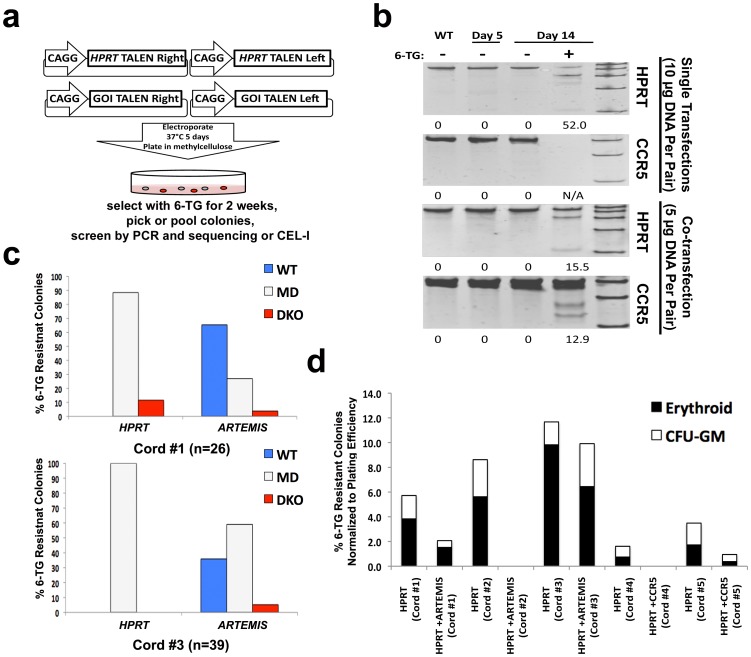
Co-targeting *HPRT* allows for robust enrichment and isolation of TALEN modified CD34^+^ cord blood progenitor cells. (a) Diagram of *HPRT* co-targeting in CD34^+^ cord blood progenitor cells. (b) Percent gene modification measured by CEL-I assay using individual *HPRT* and *CCR5* TALENs or combined using co-targeting and 6-TG selection. (c) Results of co-targeting and 6-TG selection of cells treated with *HPRT* and *ARTEMIS* TALENs using two independent cord blood samples. (d) Summary of colony formation using *HPRT* alone or co-targeting and 6-TG selection with either *CCR5* or *ARTEMIS* across 5 independent cord blood samples.

In order to develop a potentially therapeutically applicable co-targeting method for use in cells without the introduction of foreign gene sequences or induction of a transformed phenotype we chose targeting *HPRT*; cells lacking endogenous *HPRT* expression are resistant to the cytotoxic drug 6-thioguanine (6-TG)[29],[30]. Thus, we hypothesized that co-targeting *HPRT* along with another GOI could enrich and select for co-modified clones (Figure 4a). In order to test this method in a poignantly therapeutically relevant cell type, we performed *HPRT* co-targeting in CD34^+^ cord blood progenitor cells that are routinely used for hematopoietic stem cell transplants. We chose co-targeting over co-transposition to avoid the possibility of insertional mutagenesis or immune response to introduced transgenes associated with *PB* co-transposition, though we did find that *PB* transposition is functional in CD34^+^ enriched cord blood progenitors, albeit at low frequencies (>1% when normalized to plating efficiency) (Figure S8).

As TALEN modification of CD34^+^ enriched cord blood progenitors has not been previously reported, we began by targeting *HPRT* alone using nucleofection of TALEN encoding plasmid DNA and colony forming assays in methylcellulose (Figure 4a). TALEN modification was undetectable 5 days after transfection but after 2 weeks of selection in 6-TG pooled colonies demonstrated 52% gene modification (Figure 4b). Next we targeted *CCR5* and found that gene modification was undetectable at day 5, but increased to nearly 13% with *HPRT* co-targeting and selection (Figure 4b). To assess the percentage of co-modified progenitor clones using *HPRT* co-targeting, we analyzed individual 6-TG selected colonies by direct sequencing of *HPRT* and the co-targeted gene *ARTEMIS*. Sequence analysis demonstrated that 30.8% (8/26) and 64.1% (25/39) of 6-TG^R^ colonies were co-modified in two independent cord blood samples (Figure 4c). However, in a third experiment no 6-TG^R^ colonies were obtained upon co-targeting, even though targeting *HPRT* alone in this cord produced a robust number of 6-TG^R^ colonies. In fact, the generation of 6-TG^R^ colonies was highly variable with *HPRT* alone (2.2%±2.9 CFU-GM and 2.1%±2.1 erythroid) and co-targeting (4.3%±3.6 CFU-GM and 1.9%±0.75 erythroid) from individual cords (Figure 4d). However, these data demonstrates that co-targeting *HPRT* is an effective method for enrichment of TALEN modified cells in CD34^+^ enriched cord blood progenitors.

## Discussion

We have presented two methods for enrichment and isolation of nuclease modified cells based on co-transposition using the *piggyBac* (PB) transposon system and co-targeting using phenotypic selection after targeted knock out of endogenous genes. Co-transposition is a simple and efficient method for enrichment and isolation of nuclease modified cells that will likely increase the ease at which researchers can generate KO cell lines. Co-transposition is superior to previously reported enrichment and isolation methods that rely on fluorescence based surrogate nuclease reporter plasmids or having the nucleases linked physically or transcriptionally to a fluorescent protein in some manner[11],[31]. Co-transposition does not require the use of a FACS machine, which avoids exposing modified cells to high levels of hydrostatic pressure and intense lasers, recently reported to reduce viability of nuclease modified cells[13],[31]. Another enrichment method that has been recently reported uses a surrogate reporter plasmid harboring the mRFP cDNA followed by the nuclease of interests target site up stream of an out of frame H-2K^k^ or hygromycin resistance gene[13]. When the surrogate reporter is repaired, after induction of a nuclease induced DSB, the downstream out of frame selection gene is put back in frame in some instance due to indel formation. Once in frame, the H-2K^k^ or hygromycin selection marker can be used to enrich for modified cells using magnetic bead separation or antibiotic selection, respectively. Though these non-FACS based enrichment methods are functional, a new reporter plasmid must be generated for each gene target of interest and the methods do not allow for simultaneous isolation of individual clones[13],[31].

One potential drawback of co-transposition is that there is stable integration of a transposon vector that has the potential to be an insertional mutagen. However, we believe that this is not a large concern as the use of integrating lentiviral shRNA approaches to study gene KD have become standard practice. Importantly, the integration profile of lentiviral vectors demonstrates a higher propensity to integrate near oncogenes, potentially acting as an insertional mutagen, compared to *PB* [32]. We would argue that transposon integration could be beneficial as co-transposition also offers the opportunity to concurrently engineer clones with heterologous genes of interest, such as the luciferase gene for *in vivo* imaging. Indeed we have demonstrated that this can also be done with massive, up to 30 kb tested, transposon vectors that harbor 6 genes[28]. For instance, co-transposition could potentially be used to generate stably expressing chimeric antigen receptor (CAR) transgenic T-cells, as previously demonstrated using *PB*, and concomitantly enrich for TALEN modification of genes to produce T-cells that are better tumor killers, longer lived, and less susceptible to immune evasion induced by tumors[33]. Moreover, it is conceivable that co-transposition could be combined with TAL effector guided *piggyBac* transposon integration, as recently reported [34]. This may allow for both enrichment and isolation of nuclease modified cells with the added benefit of targeted transposon integration to a safe harbor site, such as AAVS1.

Stable integration of a transposon vector also allowed for the development of the conditional rescue technique described in this manuscript. This approach has the potential to make studying loss of essential genes possible. Moreover, this method directly validates that a phenotypic change induced by TALEN mediated gene KO is due to the intended modification and not an off-target event, as gene expression can be rescued by re-expression of the target gene cDNA. Another feasible approach to create inducible KO cell lines would be to perform homologous recombination (HR) to knock in a Tet system gene trap just after the target genes promoter, such as rtTA-IRES-Puro-polyA-TRE. This would interrupt normal transcription of the target gene and allow for inducible expression of the target gene using doxycycline. However, the co-transposition conditional KO method is likely superior to this idea as *piggyBac* integration is much more efficient than targeted HR and to be functional the Tet system gene trap would have to be integrated via HR at both target gene alleles.

Our second method, co-targeting, is a highly versatile method for enrichment and isolation of nuclease modified cells that can be customized for many different application of research and potentially gene therapy. We demonstrated the use of anchorage independent growth selection by targeting *PTEN* in immortalized human Schwann cells, which can be multiplexed by use of TALENs targeting other genes along with *PTEN*. *PTEN* KO enrichment is also functional in other immortalized and transformed cell lines that do not typically form colonies in soft agar but do so with the loss of *PTEN* function. For instance, we have used immortalized human osteoblast cells (iOBs) and found that *PTEN* loss induces robust colony formation and would likely allow for enrichment of the TALEN mediated KO of other genes (data not shown). More importantly, soft agar selection exemplifies the fact that nearly any selectable phenotype achieved by co-targeting can be used to enrich for and isolate TALEN modified clones.

In an attempt to demonstrate proof of principle of co-targeting for therapeutic purposes we also demonstrated successful co-targeting of *HPRT* in human CD34^+^ cord blood progenitor cells selecting for resistance to 6-thiogaunine. We believe that targeting *HPRT* for KO is potentially a viable co-targeting method for therapeutics as *HPRT* is not an essential gene and in fact humans and mice lacking functional *HPRT* are viable[29],[30]. To our knowledge this is the first demonstration of the use of TALENs to modify CD34^+^ cord blood progenitor cells. However, zinc-finger nucleases have been used previously to modify the *CCR5* locus in CD34^+^ progenitor cells by nucleofection of plasmid DNA with mean gene modification rates of up to 17% by CEL-I assay[6],[35].

Interestingly, we were unable to detect TALEN nuclease activity at the *HPRT*, *CCR5*, or *ARTEMIS* loci with our nucleofection procedures. This result was unexpected, as all of these TALEN pairs had been previously validated in transformed cell line. This discrepancy could be due to differences in nucleofection parameters as we typically only achieved transfection efficiencies ranging from 10–30%; or perhaps the repetitive nature or large size of the TALEN proteins has a detrimental effect on their expression in this cell population. However, using co-targeting *HPRT* with 6-TG selection *HPRT* gene modification was as high as 50% and this method combined with *CCR5* TALENs produced gene modification rates of nearly 13% at *CCR5*. Thus, it is conceivable that *HPRT* co-targeting could be combined with the ZFN methods of Holt et al. to further increase rates of gene modification of the *CCR5* locus in this progenitor cell population. Further studies will be needed to optimize TALEN delivery to CD34^+^ progenitor cells by nucleofection.

In summary, co-transposition and co-targeting are simple and efficient methods for enrichment and isolation of TALEN modified cells and are likely compatible with other nuclease technologies. These methods will undoubtedly expedite generation of KO cell lines for basic research studies and hold promise to improve the success of generating therapeutic cells for treatment of genetic diseases.

## Supporting Information

Figure S1 **Transient cold shock increases TALEN gene modification.** (a) CEL-I results comparing incubation of TALEN transfected cells at 37°C with cold shock treated cells at 30°C using increasing amounts of *PTEN* TALENs.
(TIF)

Figure S2 **Sequence analysis of TALEN modified clones to determine mutation type.** (a) Target site of *FOXR2*-ATG TALENs (highlighted in blue) and an example of a double knockout (DKO) clone showing bi-allelic 8 bp deletion with sequence chromatogram demonstrating complete loss of nucleotides (top). Also shown is a near identical DKO clone where two sets of double peaks (red arrows) in the sequencing chromatogram indicate slight variation at two modified alleles of a 13 bp DKO clone (bottom). (b) Example of mutation detected clone (MD) as determined by the presence of overlapping peaks in the sequencing chromatogram just after the left TALEN binding site in the spacer region (red arrow). This demonstrates that at least one or more alleles have been modified.
(TIF)

Figure S3 **Co-transposition allows for faithful expression of integrated heterologous genes in TALEN modified clones.** (a) Fluorescence and light photomicrographs of clones treated with *PTEN* or *TP53* TALENs using co-transposition of PB-CAGG-Luciferase-IRES-EGP-PGK-Puro transposon vector. (b) Luciferase imaging of clones after addition of D-luciferin substrate demonstrating robust levels of bioluminescence.
(TIF)

Figure S4 **Co-transposition using insulated RecWay assembled vectors allows for faithful expression of 6 heterologous genes.** (a) Diagram of insulated six-gene transposon vector (i6), showing the organization of 5 fluorescent protein genes and the puromycin-thymidine kinase (Puro-TK) fusion gene. Insulator elements are located between each promoter-gene element but are not shown for simplicity. (b) Fluorescence photomicrographs of two S462-TY clones generated using *PTEN* TALENs and i6 transposon for co-transposition, demonstrating expression of all 5 fluorescent proteins. Cells are also puromycin resistant indicating appropriate expression of Puro-TK gene. (c) CEL-I results using i6 gene co-transposition demonstrating robust modification enrichment of *PTEN*.
(TIF)

Figure S5 **Co-transposition can be multiplexed and is functional in HCT116 and immortalized human Schwann cells.** (a) Multiplex *PTEN* and *TP53* TALEN co-transposition results in HCT116 cells. (b) Results of CEL-I co-transposition enrichment using *PTEN* TALENs in immortalized human Schwann cells. Immortalized Schwann cells were grown to 35 days rather than the typical 14 days as their proliferation rate is much lower than transformed cells.
(TIF)

Figure S6 **Co-transposition allows for robust enrichment and isolation of CRISPR modified cells.** (a) S462-TY cells were transfected with CAGG-Flag-hCas9 and gene specific U6-gRNA plasmids in addition to CMV-PB7 and PB-CAGG-Luciferase-IRES-EGP-PGK-Puro transposon. (b) Target sequence of gRNAs used for co-transposition analysis. (c) Cells were split at day 3 after transfection and cultured +/− puromycin for an additional 14 days, analogous to co-transposition using TALENs.
(TIF)

Figure S7 **Conditional rescue co-transposition allows for faithful induction of TR-*CCND1* expression and functional changes in KO cell lines.** (a) Western blot analysis of *CCND1* on a conditional rescue DKO clone with and without doxycycline treatment compared to the parental (P) cell line demonstrating near undetectable *CCND1* without doxycycline treatment. Note the wild type controls are not represented as only MD and DKO clones were isolated from co-transposition with the *CCND1* conditional rescue transposon. (b) Proliferation assay of DKO conditional rescue demonstrating a significantly increased rate of growth in the presence of doxycycline compared to non-treated cells (t-test). (c) Soft agar colony formation assay demonstrating significantly increased colony formation upon TR-*CCND1* expression via doxycycline treatment (t-test).
(TIF)

Figure S8 ***piggyBac* transposition is functional in CD34^+^ cord blood progenitor cells.** (a) CD34^+^ cord blood progenitor cells were Nucleofected with PB-mCAGG-DHFR:EGFP transposon vector with either CMV-PB7 or Polr2a-SuperPB transposase, or no transposase control. After 5 days of incubation cells were plated in 100 nM methotrexate (MTX) containing methylcellulose media and scored after 14 days for colony formation. (b) Results of *PB* transposition after MTX selection using two independent cord blood samples.
(TIF)

Table S1 **TALEN RVD Content and spacer length.**
(XLSX)

Table S2 **CEL-I primer sequences.**
(XLSX)
