# Supplementary figures and images for: Simple and Efficient Methods for Enrichment and Isolation of Endonuclease Modified Cells
Source: PLoS One. 2014 May 5;9(5):e96114. doi: 10.1371/journal.pone.0096114 (PMC4010432; doi:10.1371/journal.pone.0096114)

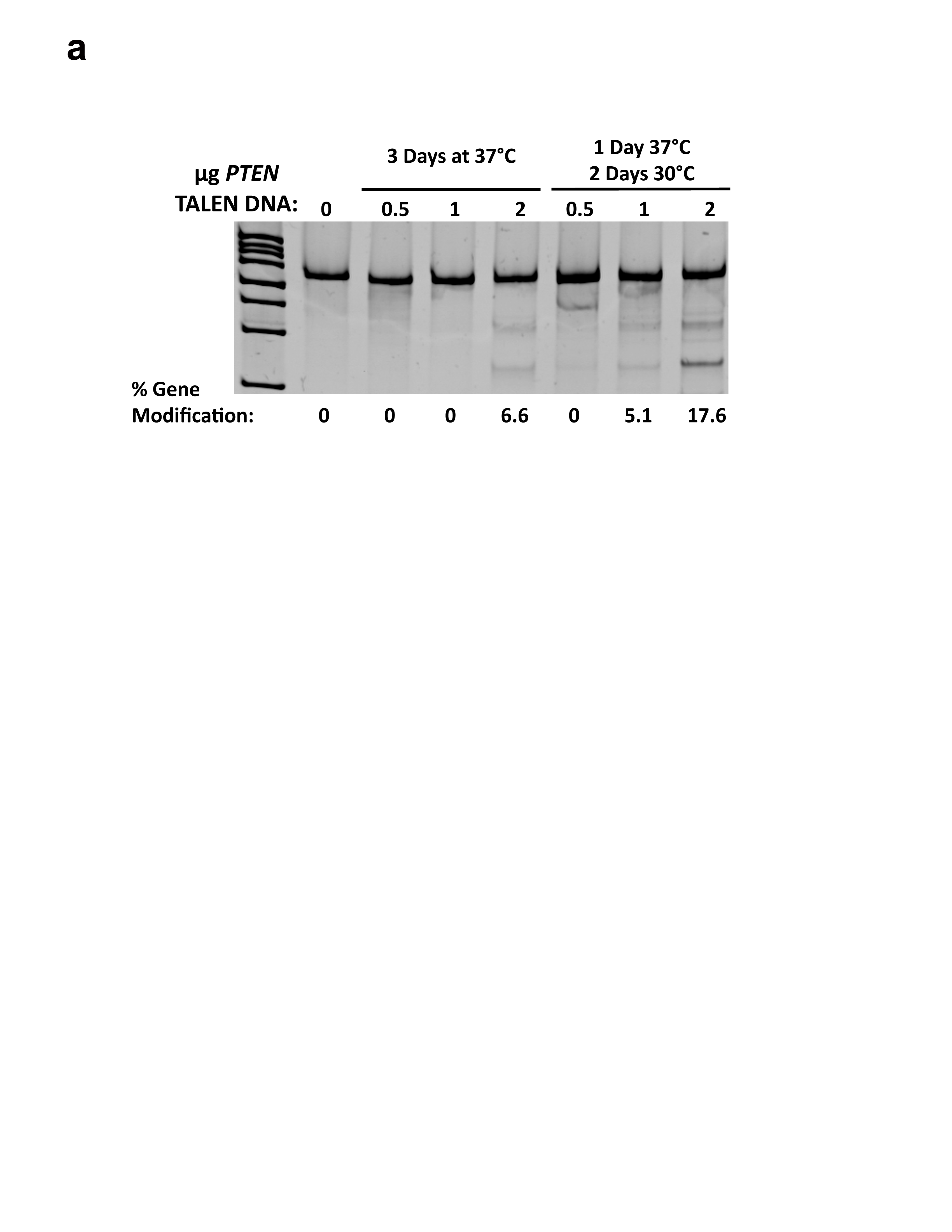

Supplement: Figure S1 — Transient cold shock increases TALEN gene modification. (a) CEL-I results comparing incubation of TALEN transfected cells at 37°C with cold shock treated cells at 30°C using increasing amounts of PTEN TALENs. (TIF) [file pone.0096114.s001.tif]

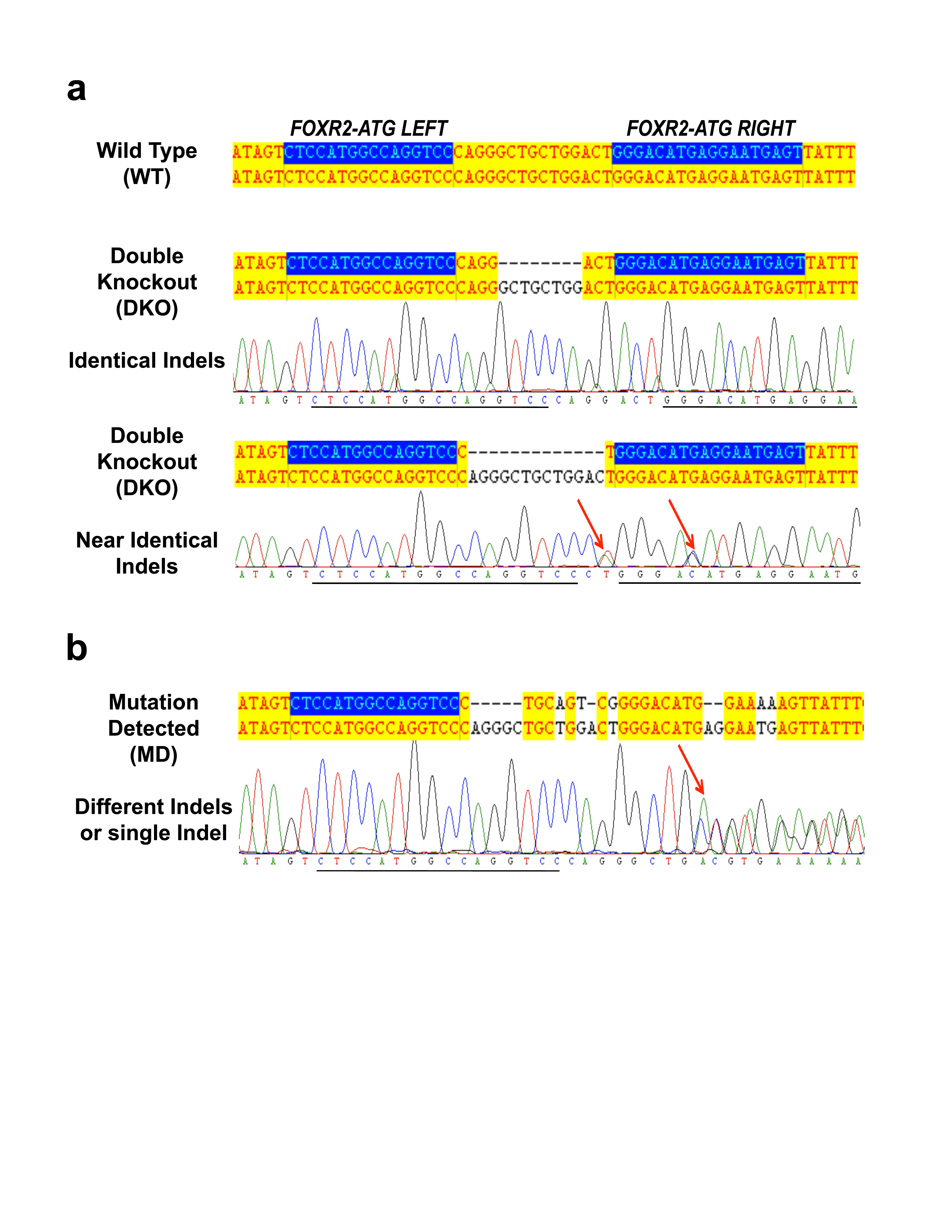

Supplement: Figure S2 — Sequence analysis of TALEN modified clones to determine mutation type. (a) Target site of FOXR2-ATG TALENs (highlighted in blue) and an example of a double knockout (DKO) clone showing bi-allelic 8 bp deletion with sequence chromatogram demonstrating complete loss of nucleotides (top). Also shown is a near identical DKO clone where two sets of double peaks (red arrows) in the sequencing chromatogram indicate slight variation at two modified alleles of a 13 bp DKO clone (bottom). (b) Example of mutation detected clone (MD) as determined by the presence of overlapping peaks in the sequencing chromatogram just after the left TALEN binding site in the spacer region (red arrow). This demonstrates that at least one or more alleles have been modified. (TIF) [file pone.0096114.s002.tif]

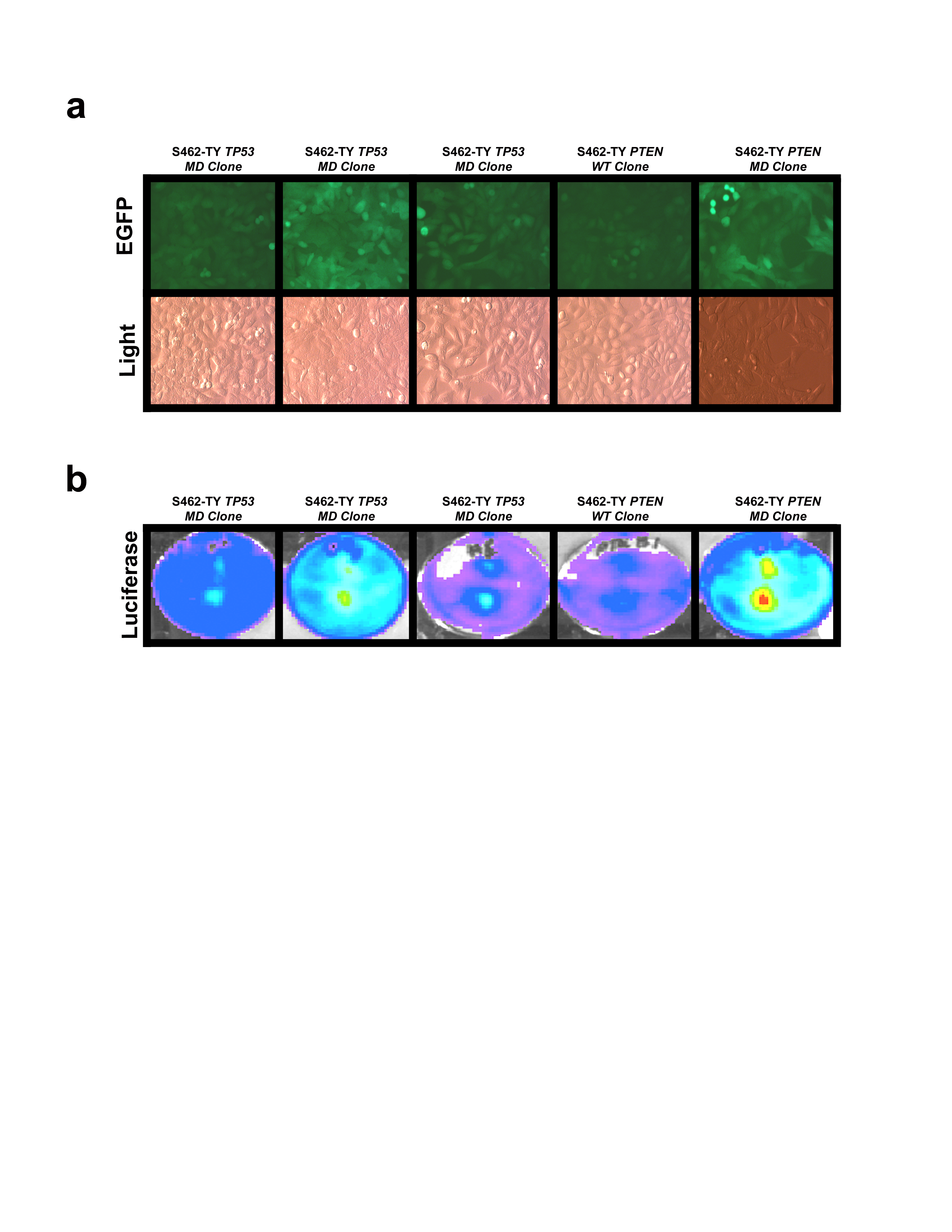

Supplement: Figure S3 — Co-transposition allows for faithful expression of integrated heterologous genes in TALEN modified clones. (a) Fluorescence and light photomicrographs of clones treated with PTEN or TP53 TALENs using co-transposition of PB-CAGG-Luciferase-IRES-EGP-PGK-Puro transposon vector. (b) Luciferase imaging of clones after addition of D-luciferin substrate demonstrating robust levels of bioluminescence. (TIF) [file pone.0096114.s003.tif]

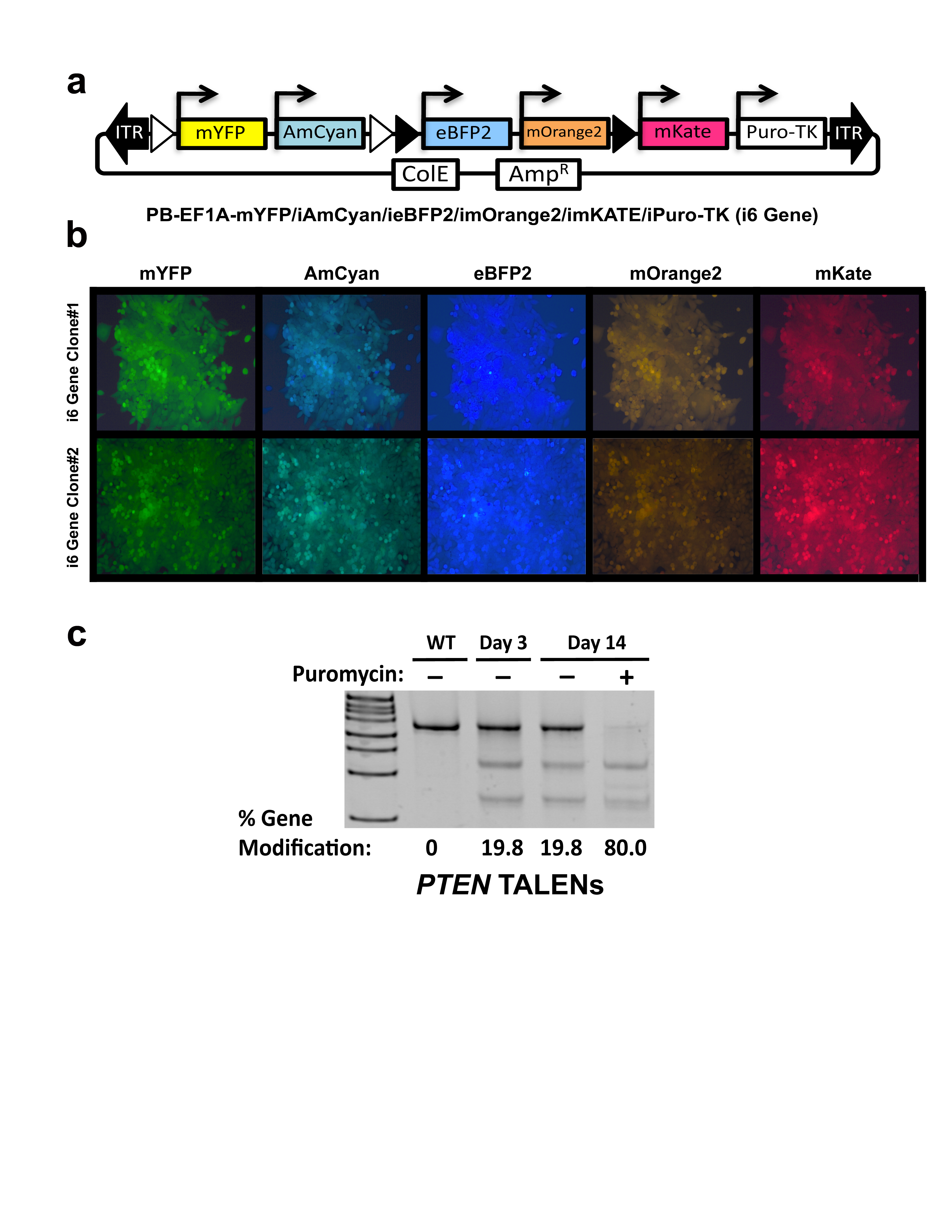

Supplement: Figure S4 — Co-transposition using insulated RecWay assembled vectors allows for faithful expression of 6 heterologous genes. (a) Diagram of insulated six-gene transposon vector (i6), showing the organization of 5 fluorescent protein genes and the puromycin-thymidine kinase (Puro-TK) fusion gene. Insulator elements are located between each promoter-gene element but are not shown for simplicity. (b) Fluorescence photomicrographs of two S462-TY clones generated using PTEN TALENs and i6 transposon for co-transposition, demonstrating expression of all 5 fluorescent proteins. Cells are also puromycin resistant indicating appropriate expression of Puro-TK gene. (c) CEL-I results using i6 gene co-transposition demonstrating robust modification enrichment of PTEN. (TIF) [file pone.0096114.s004.tif]

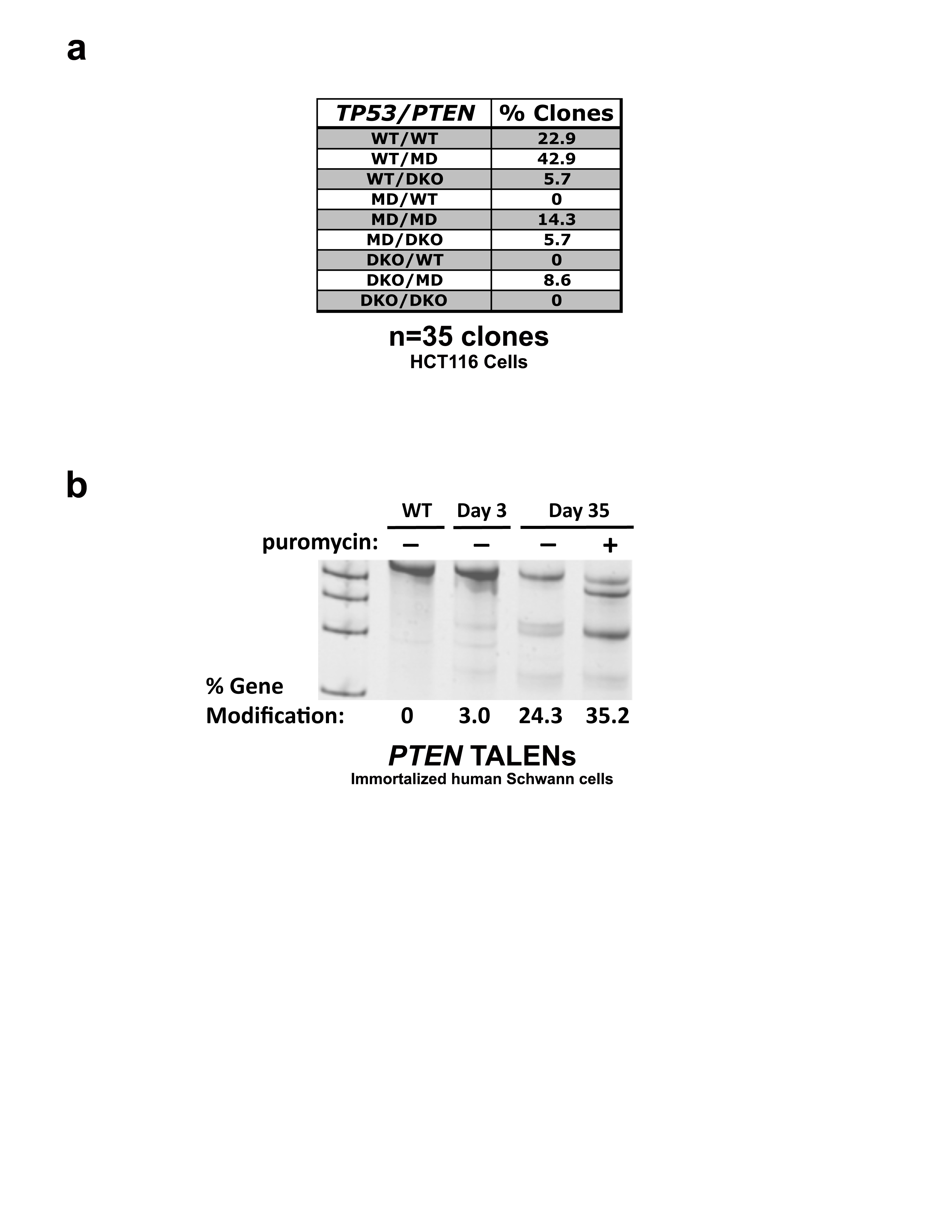

Supplement: Figure S5 — Co-transposition can be multiplexed and is functional in HCT116 and immortalized human Schwann cells. (a) Multiplex PTEN and TP53 TALEN co-transposition results in HCT116 cells. (b) Results of CEL-I co-transposition enrichment using PTEN TALENs in immortalized human Schwann cells. Immortalized Schwann cells were grown to 35 days rather than the typical 14 days as their proliferation rate is much lower than transformed cells. (TIF) [file pone.0096114.s005.tif]

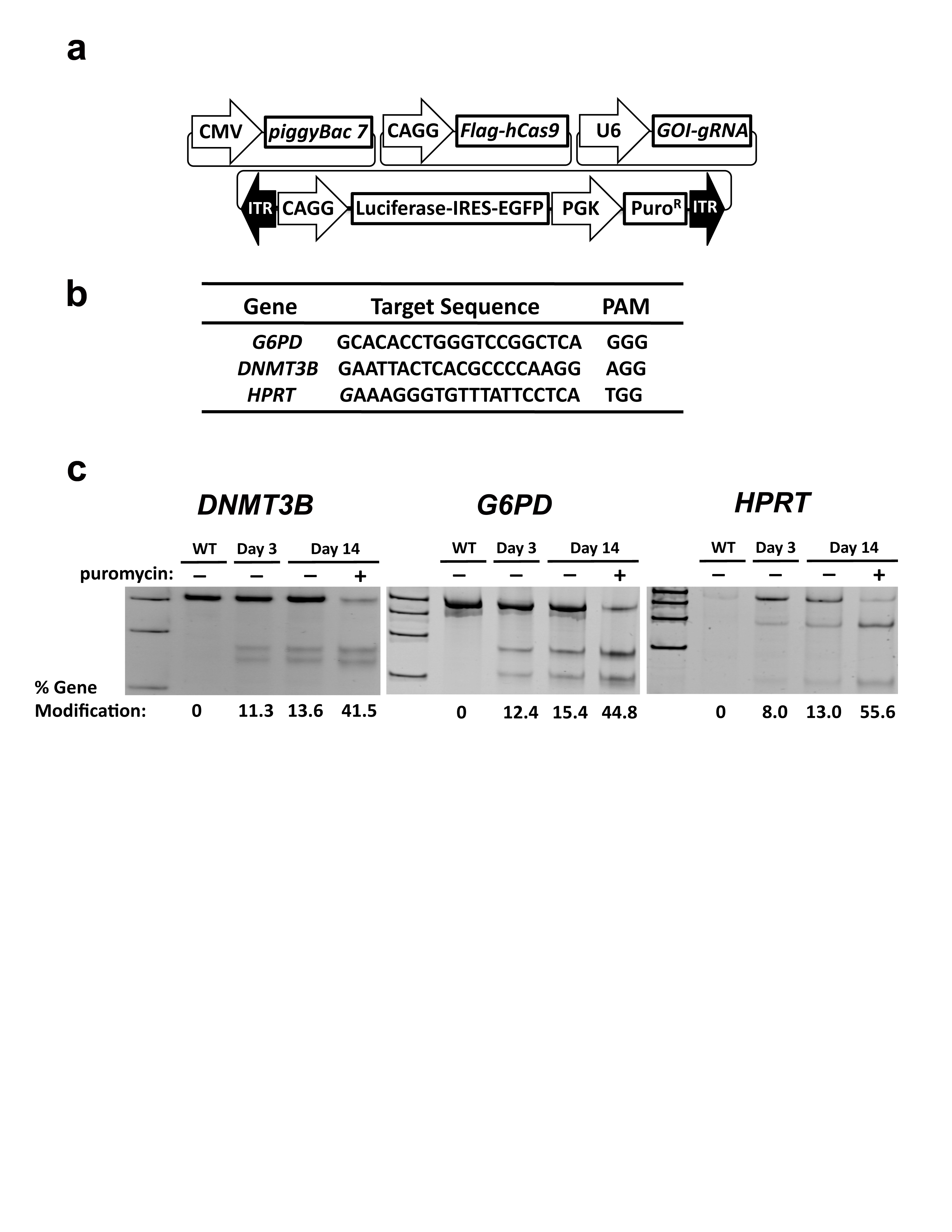

Supplement: Figure S6 — Co-transposition allows for robust enrichment and isolation of CRISPR modified cells. (a) S462-TY cells were transfected with CAGG-Flag-hCas9 and gene specific U6-gRNA plasmids in addition to CMV-PB7 and PB-CAGG-Luciferase-IRES-EGP-PGK-Puro transposon. (b) Target sequence of gRNAs used for co-transposition analysis. (c) Cells were split at day 3 after transfection and cultured +/− puromycin for an additional 14 days, analogous to co-transposition using TALENs. (TIF) [file pone.0096114.s006.tif]

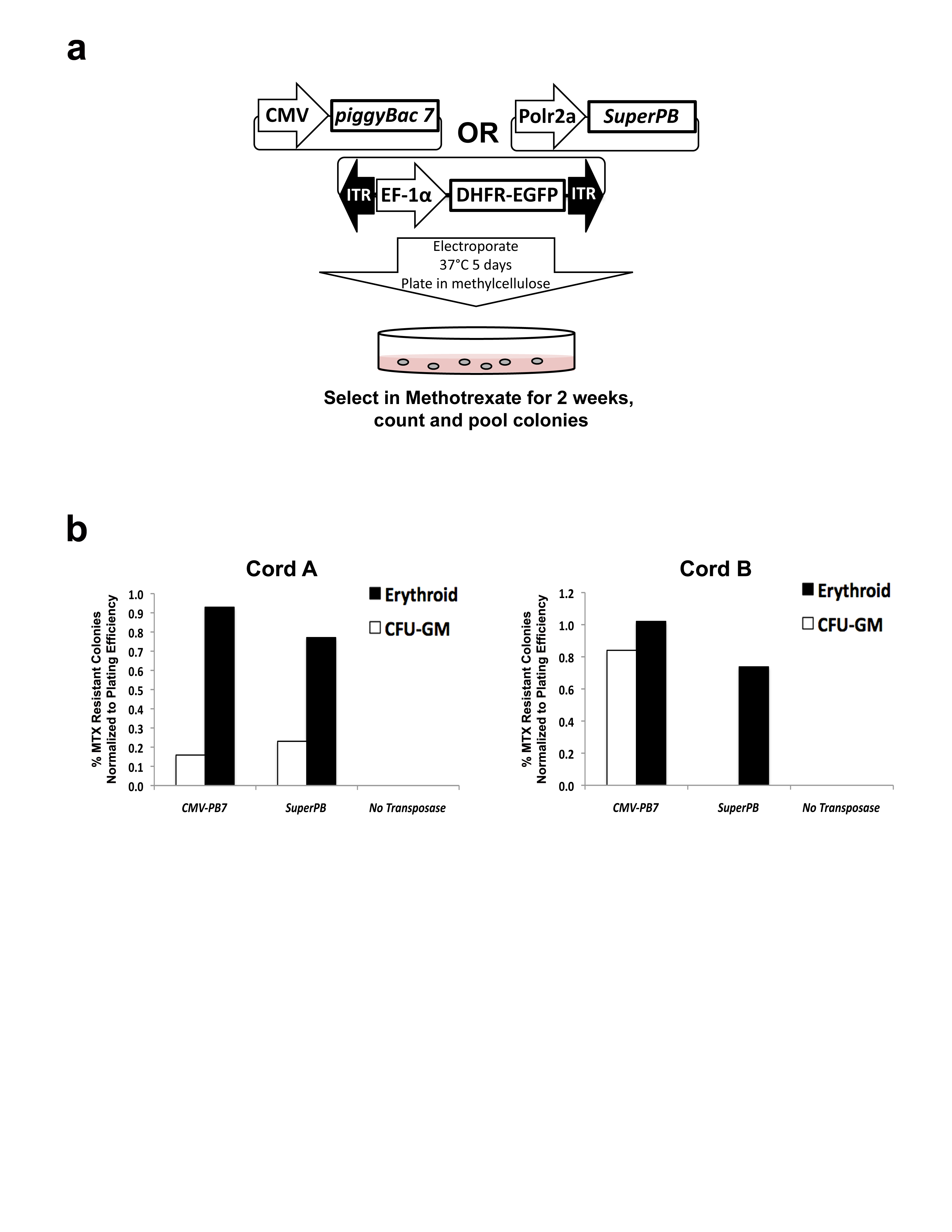

Supplement: Figure S8 — piggyBac transposition is functional in CD34+ cord blood progenitor cells. (a) CD34+ cord blood progenitor cells were Nucleofected with PB-mCAGG-DHFR:EGFP transposon vector with either CMV-PB7 or Polr2a-SuperPB transposase, or no transposase control. After 5 days of incubation cells were plated in 100 nM methotrexate (MTX) containing methylcellulose media and scored after 14 days for colony formation. (b) Results of PB transposition after MTX selection using two independent cord blood samples. (TIF) [file pone.0096114.s008.tif]
